# Predictive Value of Self-Prioritized Mobility Factors on Gait Speed and Life Space in Older Nigerians.

**DOI:** 10.1093/geroni/igaf122.2835

**Published:** 2025-12-31

**Authors:** Perpetua Obi, Isreal Adandom, Tyler Sun, Daniel Rayner, Francis Kolawole, Michael Kalu

**Affiliations:** York University, Toronto, Ontario, Canada; The University of Alabama, Tuscaloosa, Alabama, United States; McMaster University, Hamilton, Ontario, Canada; Western University, London, Ontario, Canada; University College Ibadan, Ibadan, Oyo, Nigeria; York University, Toronto, Ontario, Canada

## Abstract

Eighty-two cognitive, environmental, financial, personal, physical, psychological, and social factors significantly influence mobility decline following hospital discharge. However, assessing all these factors during the fast-paced discharge process is impractical. This study aimed to identify the factors that Nigerian older adults consider most critical and determine which factors (in combination) most realistically predict gait speed and life space among these Nigerian older adults. This is data from a cross-sectional survey that recruited 400 Nigerian older adults, 60+ years old, to rank 82 factors influencing mobility. Older adults’ gait speed and life-space mobility were collected using the 10-meter Walk Test and Life Space Assessment. Multivariate binary logistic regression was used to determine the most realistic predictor of gait speed and life-space mobility. No factors were considered critical by the older adults. The life space model indicates that increased street characteristics, social cohesion, occupation, hearing, gait speed, fear of falling, and conscientiousness accounts for approximately 50% of variations in life space. The gait speed model indicates that an increase in executive function, pain, respiratory system, body composition, fatigue, social factors, racial characteristics, marital status, social network, and fear of re-injury explain about 74% of variation in gait speed. This study provides self-reported factors that could influence older adults’ mobility following discharge that would allow clinicians to prioritize factors for assessment amidst multiple factors.

